# Effect of applied energy in renal sympathetic denervation with magnetic resonance guided focused ultrasound in a porcine model

**DOI:** 10.1186/s40349-017-0094-y

**Published:** 2017-06-12

**Authors:** Jill Shea, Joshua de Bever, Eugene Kholmovski, Hannah Beal, J. Rock Hadley, Emilee Minalga, Mohamed E. Salama, Nassir F. Marrouche, Allison Payne

**Affiliations:** 1 Department of Surgery, University of Utah, 30 North 1900 East, Salt Lake City, UT 84132 USA; 2 Department of Radiology and Imaging Sciences, University of Utah, 729 Arapeen Drive, Salt Lake City, UT 84108 USA; 3 Department of Pathology, University of Utah, 15 North Medical Drive East Ste #1100, Salt Lake City, UT 84112 USA; 4 CARMA Center, Department of Cardiology, University of Utah, 30 North 1900 East, Salt Lake City, UT 84132 USA

**Keywords:** Focused ultrasound, High intensity focused ultrasound, Renal denervation, Hypertension

## Abstract

**Background:**

Past catheter-based and focused ultrasound renal denervation studies have indicated that procedure efficacy is related to the number of ablations performed or the amount of energy used for the ablation. This study extends those prior results and investigates energy level effects on the efficacy of MR guided focused ultrasound renal denervation performed in a porcine model.

**Methods:**

Twenty-four normotensive pigs underwent unilateral denervation at three intensity levels. The applied intensity level was retrospectively de-rated to account for variability in animal size. Efficacy was assessed through evaluating the norepinephrine present in the kidney medulla and through histological analysis. The treatment was performed under MRI guidance including pre- and post-procedure T1-weighted and quantitative T1 and T2 imaging. During treatment, the temperature in the near field of the ultrasound beam was monitored in real time with MR temperature imaging. Energy delivery in the regions surrounding the renal artery was independently confirmed through an invasive fiberoptic temperature probe placed in the right renal artery.

**Results:**

Animals that underwent denervation at a de-rated acoustic intensity of greater than 1.2 kW/cm^2^ had a significantly lower norepinephrine concentration in the kidney indicating successful denervation. Images obtained during the treatment indicated no tissue changes in the kidneys as a function of the procedure but there were significant T1 changes present in the right lumbar muscles, although only one animal had indication of muscle damage at the time of necropsy.

**Conclusions:**

While MR guided focused ultrasound renal denervation was found to be safe and effective in this normotensive animal model, the results indicated the need to incorporate patient-specific details in the treatment planning of MRgFUS renal denervation procedure.

## Background

Hypertension represents a critical health challenge for millions of people throughout the world [1]. Since increased age and obesity are two of the most significant risk factors for hypertension, these numbers are expected to drastically increase [2] making the treatment of hypertension a significant public health issue. While uncontrolled hypertension in many patients is due to lack of adherence to the physician prescribed treatment, or inadequate treatment [3, 4], approximately 10% of the patient population who are currently taking three or more medications continue to have persistent high blood pressure and are identified with resistant hypertension [5].

Renal sympathetic nerves play a key role in initiating and maintaining systemic hypertension [6–8] and regulate several renal functions that are believed to contribute to hypertension [9]. Indeed, before effective pharmaceutical treatments were available, the surgical removal of these nerves was used as a treatment for hypertension [10]. The high morbidity rates of this surgical intervention led to the investigation and development of less invasive techniques including catheter-based techniques.

Recently, a radiofrequency (RF) catheter-based technique has been used to ablate the sympathetic nerves through the lumen of the main renal artery [11, 12]. While early feasibility trials showed very promising results including significant reductions in blood pressure [13], reduction of the left ventricle mass index [14], improved insulin sensitivity and glucose metabolism [15] and reduction of atrial fibrillation recurrences when used as a combination therapy [16], recent results have been more variable. The large, randomized Symplicity HTN-3 trial did not show a significant decrease in blood pressure when compared to the sham-control group [17]. There are several factors that could explain the lack of efficacy within the Symplicity HTN-3 clinical trial [18]. Approximately 40% of patients had their medications altered during the 6-month post ablation observation period and there was variability in the experience levels of the treating physicians. Both of these experimental design variables increased the probability of confounding variables [18]. Importantly, not every patient received the same number of ablation points, with subsequent data analysis suggesting that patients receiving more ablations had greater reductions in blood pressure compared with either the sham group or with patients that received fewer ablations [19]. This result indicates a potential dose effect in renal denervation procedures.

Both pre-clinical and clinical studies have demonstrated the feasibility of using focused ultrasound (FUS) as a non-invasive ablation technique to achieve renal denervation. In Wang et al. [20], healthy canines underwent bilateral renal denervation using extracorporeal FUS with ultrasound color Doppler flow imaging for targeting. When compared to the sham procedure group, a significant decrease in blood pressure (−12.3/−15.9 mmHg) and plasma noradrenaline concentration (−50.1%) were detected 6 days post-procedure. Kona Medical Company has completed two clinical trials (WAVE I [21] and WAVE II [22]) using ultrasound imaging guidance that resulted in significant 6-month blood pressure reductions (WAVE I [*N* = 24]: −29/−12 mmHg, WAVE II [*N* = 17]: −19.4/−6.5 mmHg).

Two studies have investigated performing renal denervation with MRI guided FUS (MRgFUS) in a normotensive porcine model [23, 24]. In both MRgFUS feasibility studies [23, 24], the safety of the procedure was confirmed by lack of tissue damage in the renal arterial walls as well as the skin and other intervening tissues. In [24], renal denervation was performed unilaterally in ten pigs using MRgFUS. There was a reduced kidney norepinephrine concentration in the treated kidney compared to the untreated kidney in seven of the ten animals, but no corresponding decrease in blood pressure. A signal increase in T2-weighted images indicated an increase in edema and potential damage in the treated area. In a separate study [23], a similar unilateral treatment was performed on five pigs and both safety and efficacy were assessed. A significant reduction of nerve cross-sectional area was observed as well as a reduction of kidney tissue norepinephrine concentration, ranging from a 10 to 65% decrease, indicating successful renal denervation was performed [25]. Similar to the Symplicity HTN-3 trial results, there was a positive correlation between the amount of energy applied and the reduction in kidney norepinephrine levels.

This study further investigates the potential energy level effect of renal denervation in a normotensive porcine model that has been observed in prior studies. Both the safety of the MRgFUS renal denervation procedure and the efficacy of the renal denervation procedure are reported and analyzed.

## Methods

### Animal preparation

This study was conducted with Institutional Animal Care and Use Committee approval under Good Laboratory Practices standards. Twenty-four animals (Yorkshire pigs; female; 40–50 kg) underwent unilateral renal denervation in the study. Anesthesia was induced with a Telazol, Ketamine and Xylazine cocktail (4.4, 2.2, 2.2 mg/kg respectively) administered through an intramuscular injection and maintained throughout all procedures using inhaled isoflurane (1–3%, inhaled). All hair in the intended acoustic window on the back of the animal was removed using clippers and a depilatory cream. In order to obtain an independent confirmation of energy delivery at the treatment site, a fiber optic temperature probe (Neoptix, Quebec, Canada) was placed in the right renal artery under fluoroscopic guidance through percutaneous access of the femoral artery [23]. The fiberoptic temperature probe tip was placed approximately at the bifurcation junction before entry into the kidney.

### MRgHIFU renal sympathetic denervation procedure

**Fig. 1:**
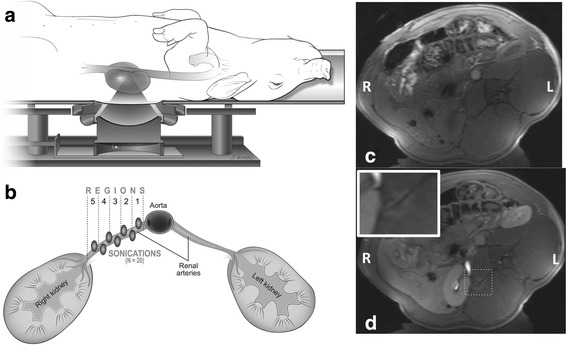
Experimental setup. **a** Schematic of animal positioned on the MRgFUS device. **b** Axial schematic of the ablation locations around the right renal artery. The segmentation of the artery into 5 regions is also shown. Axial T1-weighted MR images of the pig position are shown (**c**) pre- and (**d**) post-treatment. Some edema can be seen around the spinous process as shown in the inset in (**d**)

The renal denervation procedure was performed with a pre-clinical large animal MRgHIFU system (*f* = 1 MHz, 256-element phased-array transducer, 1.3 × 1.3 × 8 mm full-width-half-maximum focal spot size, 13 cm focal length, Image Guided Therapy, Inc., Pessac, France). The animal was placed in a rotated supine position on a custom holder with an integrated 9-channel MRI receive coil and the skin was coupled to the transducer with degassed, deionized water. The animal position was adjusted such that the renal artery was placed in close proximity of the geometric focus of the transducer while avoiding the spine in the acoustic window of the ultrasound beam. A schematic of the experimental setup is shown in Fig. 1. The experiment design had six study groups with acoustic intensity and study endpoint as variables as detailed in Table 1. The spatial-averaged-time-averaged acoustic intensity (I_SATA_) values (5.9, 10.4, 14.8 kW/cm^2^) were determined through both radiation force balance and hydrophone measurements performed in degassed water. The spatial average was taken over the full-width-half-maximum (FWHM) of the ultrasound beam as measured by hydrophone.

**Table 1:** Six study groups that underwent unilateral renal denervation with MRgFUS

Group ID	I_SATA_ (kW/cm^2^)	Study Endpoint	Number
1	5.9	9 days	6
2	10.4	9 days	6
3	14.9	9 days	6
4	5.9	6 months	2
5	10.4	6 months	2
6	14.9	6 months	2

The spatial-averaged-time-averaged intensity (*I*_*SATA*_) in water, time duration between the renal denervation and euthanasia and number of animals per group are listed

Pre-treatment imaging consisted of 3D T1-weighted Volumetric Interpolated Breathhold Imaging (VIBE), dark blood turbo spin echo (TSE), and T1-(ECG-triggered Modified Look-Locker Inversion Recovery sequence, [26]) and T2-(T2-prepared TrueFISP sequence, [27]) mapping. Typical scan parameters used in all these sequences are shown in Table 2.

**Table 2:** Typical scan parameters for all MRI sequences used in the study

Sequence	TR/TE (ms)	Flip angle	# of slices	Matrix size	Resolution (mm)	Orientation
3D T1w Vibe	4.3/2	12°	56	154 × 320	1.7 × 1.2 × 3	Axial
T2w TSE	2000/89	180°	17	202 × 256	1.4 × 1.3 × 4	Axial
MOLLI, T1-map	2.6/1.1	35°	1	124 × 192	2.2 × 1.8 × 5	Axial
TrueFISP, T2-map	2.7/1.1	38°	1	126 × 192	2.2 × 1.8 × 5	Axial
3D segmented-EPI, MRTI	30/8	20°	10	96 × 128	2 × 2 × 3	Coronal

All animals received approximately 20 individual sonications in the regions immediately surrounding the right renal artery (±5 mm) using the acoustic intensity indicated in Table 1. Because imaging artifacts due to the presence of motion (e.g. blood flow, peristalsis, respiration) and fat render MR temperature imaging (MRTI) techniques inaccurate in the regions directly surrounding the renal artery, MRTI measurements were not obtained in the treatment region. However, MRTI was performed in the near-field regions surrounding the spine during all MRgFUS sonications in real time using a 3D segmented Echo Planar Imaging (EPI) sequence (parameters detailed in Table 2). The temperature measurements obtained from the fiber optic probe placed in the right renal artery were also recorded continuously throughout the denervation procedure. The mean electric power output was recorded for every sonication; acoustic power output was later determined using force-balance measurements obtained outside the MRI [28]. After the renal denervation procedure, the pre-treatment imaging protocol was repeated. In addition, post-contrast 3D T1w VIBE scans were performed after injection of contrast agent (0.1 mmol/kg of MultiHance, Bracco Diagnostics Inc., Princeton, NJ) to evaluate the tissues surrounding the treatment region.

### Tissue harvest and preparation

At the time of harvest the renal artery, kidneys, and underlying muscle were examined bilaterally for any gross abnormalities. Subsequently, the kidneys were removed, and a segment of the medulla was dissected and frozen immediately on dried ice. This tissue was used to evaluate levels of norepinephrine (see methods below). The renal artery and surrounding tissue were harvested and fixed in formalin for a minimum of 24 h. Post fixation, the length of the left and right arteries were measured and then each artery divided into five equal segments. Region 1 was designated as the region closest to the aorta and region 5 closest to the kidney as indicated in Fig. 1. The segments were dehydrated in increasing concentrations of alcohol, embedded in paraffin, sectioned (5 μm), and then stained with haematoxylin and eosin (H&E) or Masson’s Trichrome Stain.

### MRI data analysis

**Fig. 2:**
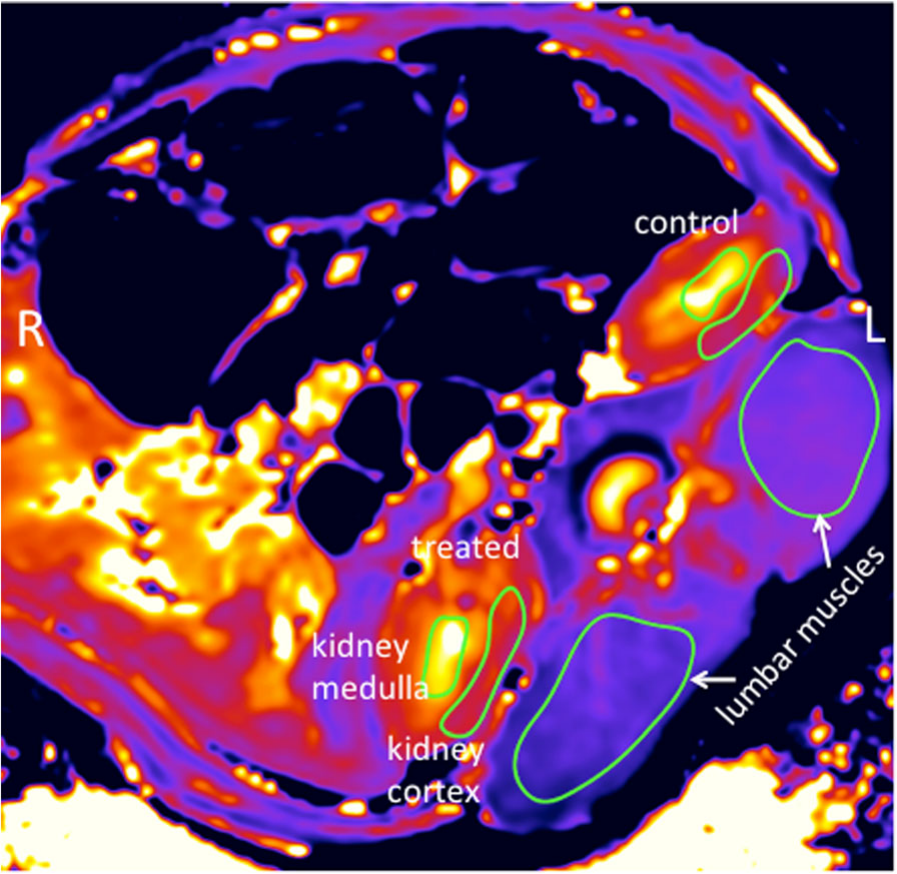
Pre-treatment T1-map obtained in an animal from study group 1. The masks used for calculating the mean T1 values are shown in the lumbar muscles as well as the treated and control kidney medulla and cortex

Temperature measured in the near-field of the ultrasound beam was analyzed for every sonication. Any tissue changes in the insonified regions due to the procedure were identified through comparison of the pre- and post-treatment MRI images. In the post-contrast 3D T1w VIBE images tissue changes around the spinous process were quantified through measuring the longest dimension of any resulting hyperintense regions. In addition, the pre- and post-ablation T1- and T2-maps were quantified in the kidney medulla and cortex as well as the lumbar muscle tissue to determine if any tissue changes had occurred as a result of the MRgFUS renal denervation procedure. T1 and T2 quantification was performed by manually identifying masks in the appropriate tissue regions (Fig. 2) and recording the mean value in that tissue region. While equivalent masks were used for the T1- and T2-maps for the same animal, the inter-animal mask placement and size varied depending on the animal’s anatomy. The left lumbar muscle, which was not in the ultrasound field, served as a control in order to verify the accuracy of the T1 and T2 maps through the animal. If the pre- and post-treatment T1 or T2 mean value in the left muscle varied more than ±5%, those data were not included in the final analysis.

### Morphometric analysis

One H&E section from each renal artery region was histomorphometrically evaluated via light microscopy. In addition, H&E stained slides were digitally scanned using a ScanScope® AT2 system. The ImageScope software in eSlideManager (Aperio/Leica Biosystems, Vista, CA) was used to visualize each slide [23, 29]. Quantitative measurement tools including positive pixel count algorithms within the ImageScope software were used to determine the number of nerves, nerve cross-sectional area, arterial area, and the distance between the arterial lumen and the nerve. For calculation and analysis of mean nerve area, only nerves with an area greater than 5000 μm^2^ and smaller than 70,000 μm^2^ were included in the calculation. Nerve area reduction results are presented as a ratio of the summation of the treated to control sides for each animal (*Area*_*ratio*_ = *Area*_*treated*_/*Area*_*control*_).

### Pathological review

A pathologist blinded to the group assignments evaluated the H&E and Masson’s trichrome stained histological sections to determine if there was any evidence of nerve or arterial damage in the form of necrosis, inflammation, fibrosis, hyperplasia, or structural abnormalities. Arterial wall was evaluated for evidence of fibrinoid necrosis, concentric thickening, attenuation, hyalinizaytion, edema, inflammation or collagen deposition. In addition, for each artery lining endothelium was evaluated for evidence of sloughing, necrosis, hyperplasia or evidence of thickening in basement membrane. Nerve tissue was evaluated for evidence of traumatic or degenerative changes.

### Norepinephrine analysis

At the time of analysis the frozen kidney sections prepared during the necropsy were thawed, weighed, and then homogenized in ice cold 0.8 M EDTA (Ethylenediaminetetraacetic acid). The concentration of norepinephrine within the homogenate was determined with an enzyme-linked immunosorbent assay (ELISA) following the manufacturers instructions (Rocky Mountain Diagnositcs, Colorado Springs, CO). Norepinephrine results are presented as a ratio of the concentration measured in the treated to the control kidney (*NE*_*ratio*_ = *NE*_*treated*_/*NE*_*control*_).

### Power adjusted analysis

In all study groups, the energy level was applied independent of animal size and anatomy. In order to retrospectively account for the variation in animal size during data analysis, animals were re-grouped into two populations based on a de-rated spatial-averaged-time-averaged acoustic intensity (*I*_*D*_) defined in Eq. 1

1 $$ {I}_D= I{e}^{-2\alpha z} $$

where *I* is the acoustic intensity in water, *α* is an assumed homogeneous attenuation value 0.15 Np/(cm*MHz) for soft tissue [30], and *z* (cm) is the mean ultrasound beam propagation path length from the animal skin to the ablation site. *I*_*D*_ and *z* are shown for each animal in Table 3. For analysis, animals were regrouped by endpoint as receiving either a low (<1.2 kW/cm^2^) or high (≥1.2 kW/cm^2^) *I*_*D*_.

**Table 3:** Summary of study parameters. Time to Evaluation (TTE) was either 9 days (9d) or 6 months (6 m)

Animal ID	TTE	Acoustic Power (W)	Mean propagation path length, *z* (cm)	Derated Intensity, *I*_*D*_ (kW/cm^2^)	Medulla norepinephrine (ng/mL/g)			Spinous process enhancement (mm)
					Left	Right	Ratio (R/L)	
11	9d	80	8.9	0.40	2322	1322	0.57	0
2	9d	80	8.6	0.41	1131	1480	1.31	0
9	6 m	80	7.5	0.62	1655	2440	1.47	0
12	9d	80	7.3	0.65	2402	1187	0.49	20
20	9d	80	7.2	0.67	1658	3239	1.95	0
18	9d	80	7.1	0.70	2248	1334	0.59	0
5	6 m	80	7.1	0.74	591	1241	2.10	0
17	9d	80	6.5	0.81	810	771	0.95	21
8	9d	200	9.5	0.90	992	1139	1.15	19
7	6 m	140	7.8	1.00	3048	2907	0.95	0
14	9d	140	7.7	1.04	484	789	1.63	18
1	9d	140	7.5	1.10	899	2572	2.86	9
21	9d	140	7.5	1.11	855	892	1.04	20
3	9d	200	8.0	1.19	992	2246	2.26	7
19	9d	140	7.0	1.31	4478	1350	0.30	28
4	6 m	140	6.9	1.33	229	29	0.12	19
15	9d	200	8.0	1.38	861	953	1.11	38
13	9d	200	7.7	1.49	1880	1216	0.65	20
6	6 m	200	7.3	1.49	646	295	0.46	0
16	9d	140	6.4	1.54	539	385	0.71	17
23	9d	140	6.3	1.54	1027	197	0.19	11
24	9d	200	7.6	1.59	1339	837	0.63	22
10	6 m	200	7.3	1.70	1065	1457	1.37	0
22	9d	200	7.2	1.78	653	311	0.48	27

Spinous process enhancement measures the maximum length of any hyper intensity present around the right spinous process after the post-contrast 3D T1w VIBE images. The solid line separator indicates the animal treated with a low or high derated acoustic intensity (1.2 kW/cm^2^ threshold)

### Statistics

To determine if there was a differential response between the low and high rated energy groups, kidney norepinephrine and nerve area ratios were compared with a *t*-test (JMP Pro 11; SAS; Cary, NC) with significance set at *p* < 0.05.

## Results

**Fig. 3:**
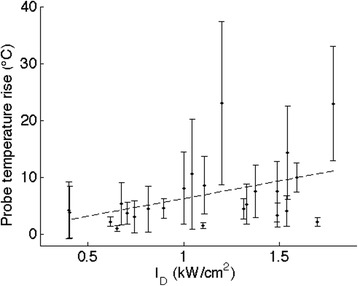
Mean temperature rise measured by the intravascular fiber optic probe as a function of de-rated spatial-averaged-time-averaged acoustic intensity (*I*_*D*_) for each animal. The mean was computed over all sonications. Error bars indicate the standard deviation over all sonications for a single animal

All animals tolerated the procedure with no adverse events. No skin burns or irritations were noted and all animals recovered with minimal analgesic support (single dose 4 mg/kg Carprofen, intramuscular delivery). Energy delivery to the treatment region was confirmed through the use of an invasive fiber optic temperature probe inserted into the right renal artery. The mean temperature rise detected in the treatment region by the probe as a function of de-rated power is shown for all animals in Fig. 3. The mean is computed over all sonications performed in each animal. A weak increasing linear trend of increased temperature rise with applied power is observed (R^2^ = 0.19, *p* = 0.03).

**Fig. 4:**
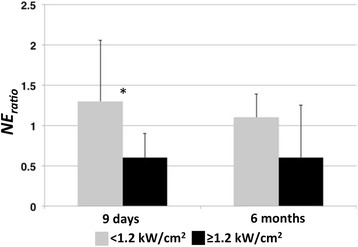
Kidney medulla norepinephrine ratio (NE_ratio_) as a function of low (<1.2 kWcm^2^) and high (≥1.2 kWcm^2^) de-rated acoustic intensity. Error bars indicate one standard deviation. *The difference between groups was found to be significant (*p* = 0.01)

Efficacy of the procedure was determined by comparing the ratio of the treated to control group for both nerve area (*Area*_*ratio*_) and kidney medulla norepinephrine (*NE*_*ratio*_). As shown in Fig. 4, at 9 days post-renal denervation procedure the *NE*_*ratio*_ in the group with *I*_*D*_ ≥ 1.2 kW/cm^2^ was significantly lower when compared with the group with *I*_*D*_ <1.2 kW/cm^2^ (0.6 ± 0.3 vs 1.3 ± 0.8, *p* = 0.01), however the *NE*_*ratio*_ reduction after 6 months was not significant (0.6 ± 0.7 vs 1.1 ± 0.3, *p* = 0.3). There was no difference in *Area*_*ratio*_ between the low and high *I*_*D*_ groups at 9 days (low: 0.98 ± 0.3 vs. high: 0.88 ± 0.27, *p* = 0.43) or 6 months (low: 1.1 ± 0.3 vs. high: 0.9 ± 0.2, *p* = 0.3) post-renal denervation procedure.

**Fig. 5:**
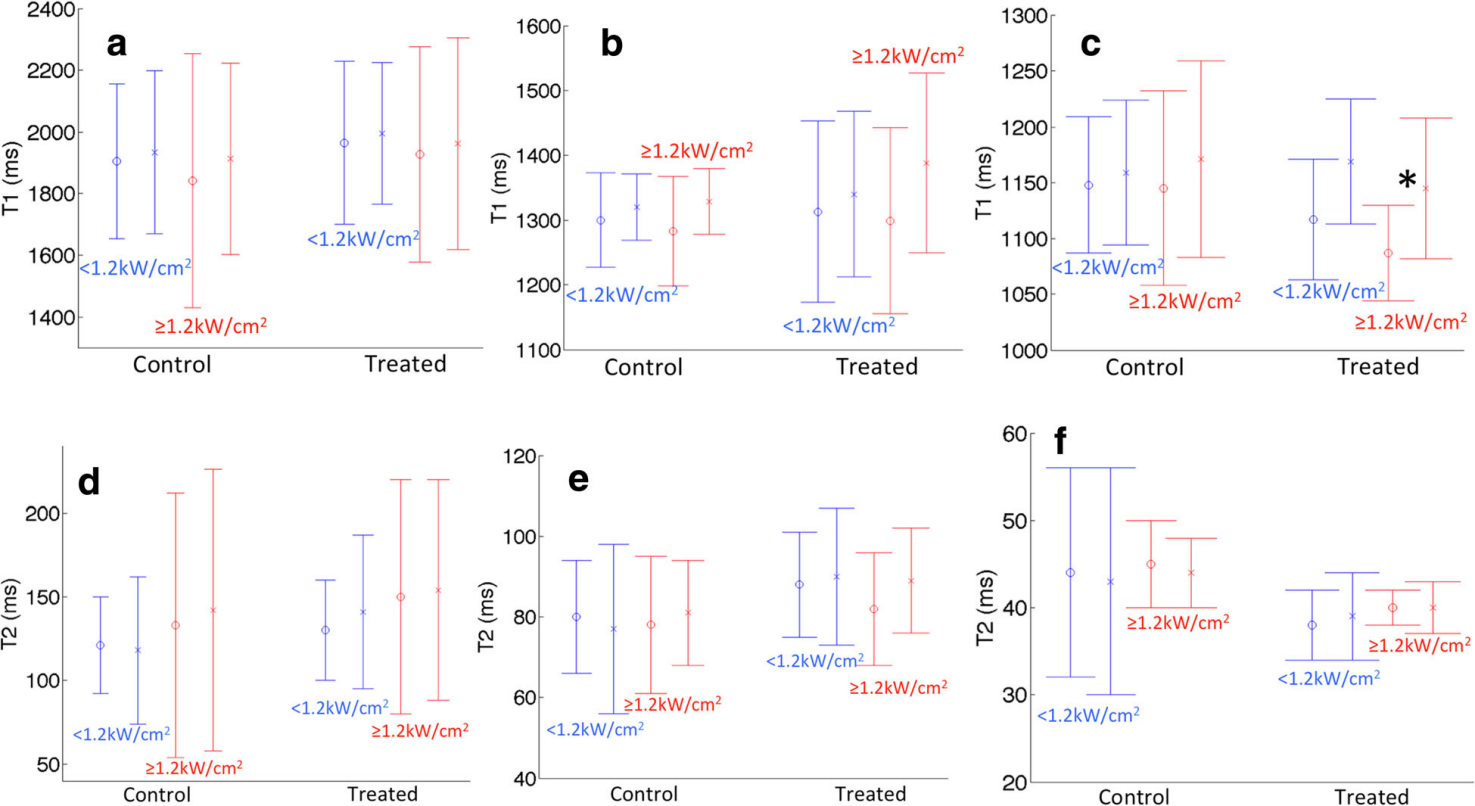
Mean T1 (**a**–**c**) and T2 (**d**–**f**) values for the treated and control sides of the (**a**, **d**) kidney medulla, (**b**, **e**) kidney cortex and (**c**, **f**) lumbar muscle tissues. The mean values are computed for animals in the low (<1.2 kWcm^2^) and high (≥1.2 kWcm^2^) de-rated acoustic intensity groups. For each group the pre- and post-treatment values are displayed. Error bars represent one standard deviation. Significant differences between pre- and post-treatment groups are denoted by *

**Fig. 6:**
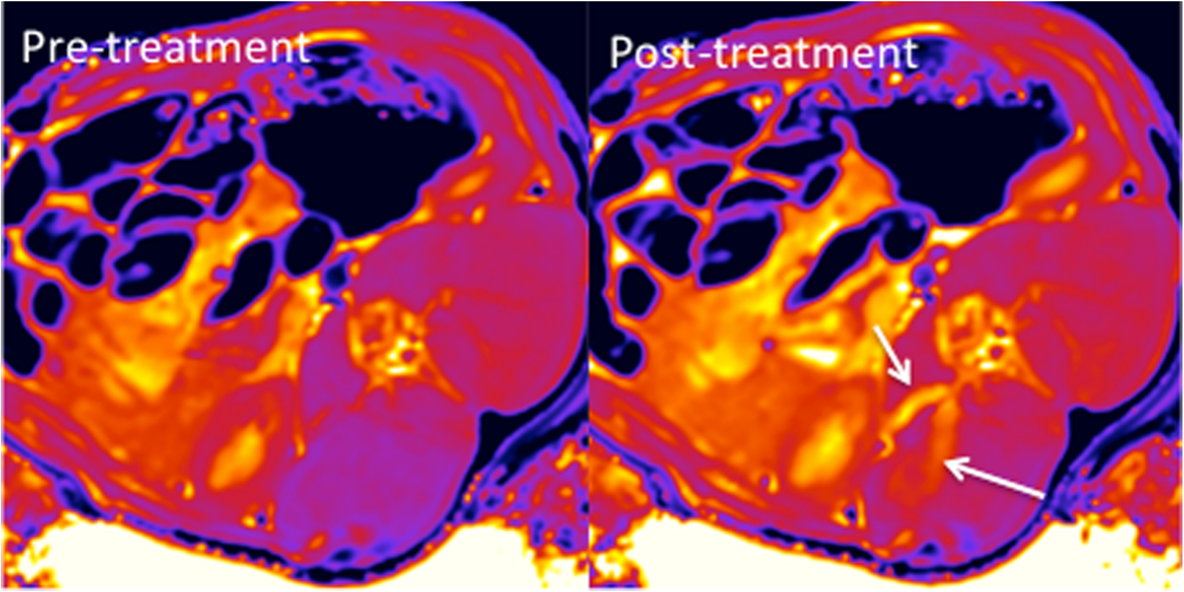
Pre- and post-treatment T1-maps obtained in animal 19 in study group 2 (10.4 kW/cm^2^, 9 day endpoint). Increased edema in the right lumbar muscle and around the spinous process is seen in the post-treatment image as indicated by the *arrows*

Quantitative pre- and post-renal denervation procedure T1 and T2 maps were acquired in 17/24 of the animals on study. For the seven animals with incomplete data, T1- and T2- maps could not be obtained for three of the animals due to MRI scheduling limitations and hardware difficulties. In the four other animals, the data was obtained but the variance between pre- and post-treatment measured T1 and/or T2 values in the left lumbar muscle outside of the acoustic window were larger than ±5% indicating the presence of artifacts or other scanning issues and the data was therefore excluded from analysis. For all other data sets, there was no significant difference in the measured T1 and T2 values in the kidney medulla and cortex when comparing both the treated and control kidney both pre- and post-renal denervation treatment. As shown in Fig. 5, there was no significant difference between the low (<1.2 kW/cm^2^) or high (≥1.2 kW/cm^2^) *I*_*D*_ groups. There was a significant difference in the measured T1 value in the right lumbar muscle, located in the near field of the acoustic window (*p* = 0.03). This difference is due to the edema present after the ablation procedure in 15/24 of the animals (mean length = 19.75 ± 7.7 mm) as reported in Table 3 and shown for animal 19 in Fig. 6.

**Fig. 7:**
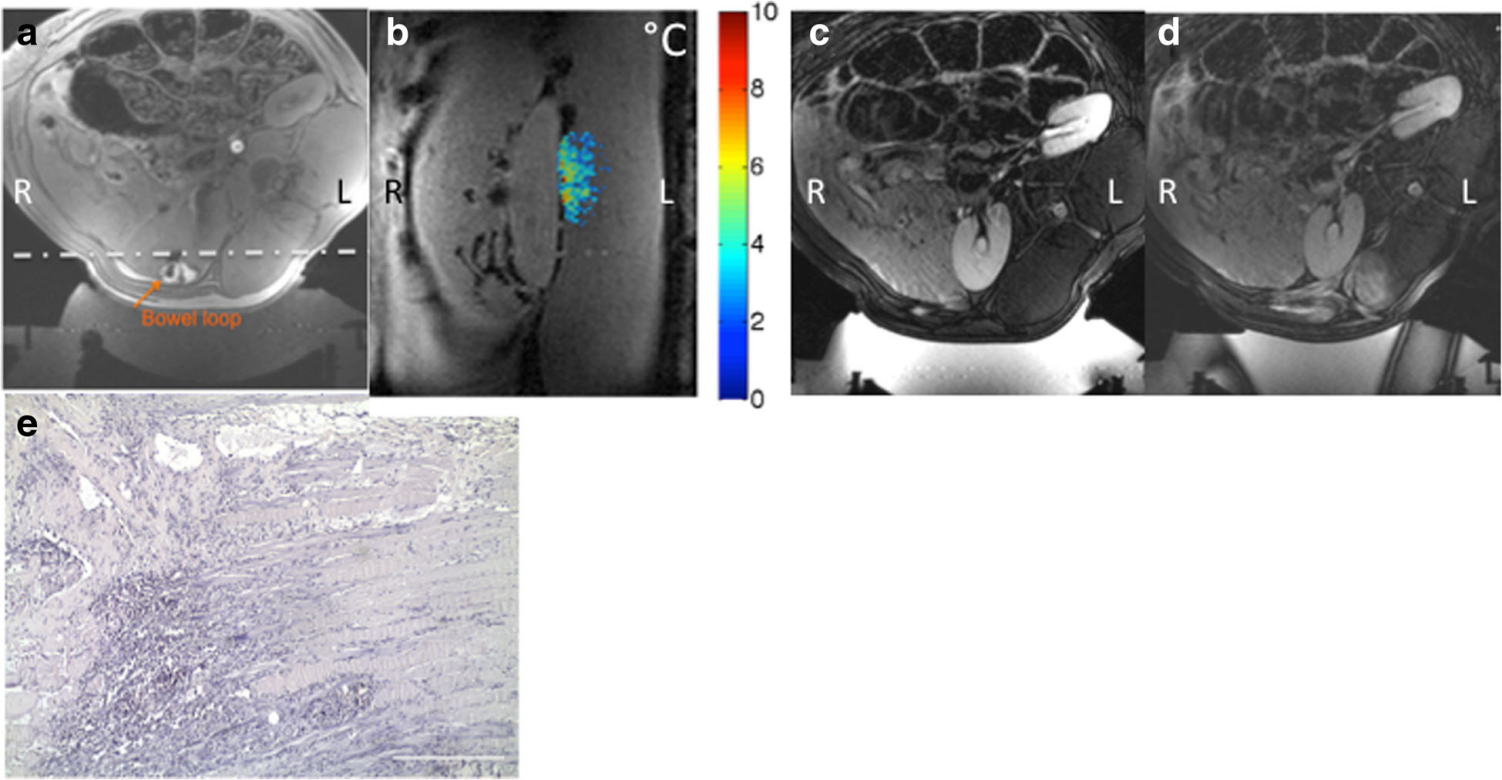
Results of animal ID 8, study group 3. **a** An air-filled bowel loop was present in the near-field of the ultrasound beam. **b** Coronal MR temperature image obtained during one ablation point showing near-field heating. Slice position is indicated by the *dashed line* in (**a**). **c** Pre- and **d** post-treatment T1w images clearly show the resulting extensive tissue changes in the near field. **e** H&E slide of the muscle tissue shows muscle damage was present at the time of necropsy

One animal (animal ID 8, study group 3) did have muscle damage present at the time of necropsy. In this animal, an air-filled bowel loop was adjacent to the lateral side of the right kidney and was present in the acoustic window as seen in Fig. 7(a). This caused reflections and interference with the ultrasound beam resulting in thermal energy accumulation as measured by the real time MR thermometry. A representative thermal map obtained during one sonication is shown in Fig. 7(b). The accumulation of thermal damage over the twenty sonications performed resulted in tissue injury and extensive edema throughout the intervening tissues including the muscle and fat. A comparison of pre- and post-treatment T1w images are shown in Fig. 7(c) and (d). A non-enhancing region (1 × 0.8 cm) surrounded by enhancement was present in the muscle in the post-procedure delayed contrast enhanced images acquired 10 min after the contrast injection was administered. This was confirmed by H&E histologic analysis as shown in Fig. 7(e).

After sacrifice, the excised renal artery was measured and then divided into five approximately equal segments. The length of the renal artery on the treated side was statistically longer than the control side at 9 days (treated: 30.4 ± 4.6 mm vs. control: 33.4 ± 5.6 mm, *p* < 0.01) but not at 6 months (treated: 33.4 ± 5.6 mm vs. control 29.6 ± 4.0 mm, *p* = 0.13). There was no difference in renal artery area between the treated and control sides at either the 9 day (treated: 4.2 ± 1.9 mm^2^, control: 4.1 ± 1.7 mm^2^, *p* = 0.96) or 6 month (treated: 8.8 ± 2.4 mm^2^, control: 7.9 ± 1.9 mm^2^, *p* = 0.45) study endpoint. In terms of nerve histology, 82% of the nerves on the treated side and 79% of the nerves on the control side were located within less than 3 mm of the lumen of the artery. Additionally, 68% of the nerves on the treated side and 71% of the nerves on the control side were located between Regions 3–5 (Fig. 1), closer to the renal pelvis.

### Pathological review

One animal (animal ID 8, study group 3) did have muscle damage present at the time of necropsy as shown in Fig. 7(e). This was predominantly composed of chronic lymphocytic infiltrate of the muscle tissue. Histopathologic review indicated no evidence of tissue damage involving artery wall endothelium, artery wall, or nerve tissue of any of the animals tested. In addition there was no difference observed between control groups at 9 days, or 6 month in comparison to their corresponding time point treated animals either in the high or low adjusted intensity animal groups.

## Discussion

This study complements previous work that indicated RF catheter and focused ultrasound renal denervation procedures are more efficacious with increased energy deposition [23] or number of ablations [17]. While this MRgFUS renal denervation study evaluated three distinct applied acoustic intensities, a retrospective analysis scheme was employed to take into account the inter-animal variance of the distance between the skin and target region (6.3 to 9.5 cm). A de-rated acoustic intensity metric was computed for each animal by simplifying the heterogeneity of the animal anatomy to a homogeneous medium with an assumed attenuation value of muscle. When the animals were regrouped using this metric, it was found that animals with a de-rated acoustic intensity of ≥ 1.2 kW/cm^2^ had a significantly lower kidney medulla norepinephrine ratio (*NE*_*ratio*_), reduced by approximately 54%, 9 days after the renal denervation procedure. Since kidney medulla norepinephrine is a proven robust marker for effective renal nerve destruction [25], this significant decrease further confirms that increased energy deposition leads to more efficacious outcomes. However, unlike a previous study [23], a reduction of nerve cross-sectional area was not observed in this study. In addition, there was not a consistent demonstration of nerve damage as measured through histologic analysis. Therefore, while there was a significant decrease in NE seen in the higher de-rated acoustic intensity group, this efficacy was not confirmed by a secondary metric.

The retrospective de-rated acoustic intensity metric used in this study also highlights the need to incorporate patient-specific details when developing a treatment plan for renal denervation FUS procedures. Due to the difficulty of obtaining MR temperature imaging data at the treatment region immediately surrounding the renal arteries, it is difficult to calibrate applied power in situ with a test sonication as is done in other clinical MRgFUS procedures [31]. Freyhardt et al. [24] did have a calibration step in their porcine renal denervation study. Test sonications were performed in the ipsilateral longissimus lumborum muscle, posterior to the target site. However, the energy was determined based on a uterine fibroid protocol and was not tailored specifically to renal denervation. The use of a subject-specific derating metric that took into account the heterogeneity of each subject would potentially increase the efficacy of the denervation procedure.

As demonstrated by the excessive near-field heating in animal 8, the incorporation of additional patient-specific details for renal denervation treatments must include the presence of bone and bowel in the acoustic window. This has been shown in other MRgFUS clinical applications such as uterine fibroid treatments where segments of bowel anterior to the target area can adversely affect the MRgFUS treatment [32]. The gas present in the bowel causes the ultrasound energy to be reflected towards the skin causing unwanted heating away from the intended target. While techniques can be employed to mitigate these effects, such as rectal filling, these techniques were not used in this study.

It has been demonstrated that phase aberration can affect the efficiency of an ultrasound treatment not only in transcranial applications [33] but also in soft tissue anatomies, such as the breast [34, 35]. For renal denervation applications, the presence of the spine as well as multiple tissue types in the acoustic window (including muscle, fat, kidney and liver) can potentially distort the ultrasound beam’s focus and reduce the energy delivered to the treatment region. The variability of the temperature rise measured with the intravascular fiberoptic temperature probe as a function of de-rated power could be due to the presence of phase aberrations. While not included in this work, this hypothesis is currently being further evaluated by a study that is retrospectively simulating all the renal denervation treatments in this work by creating segmented models incorporating all tissue types and performing ultrasound simulations.

In this study, approximately 70% of the nerves were located in regions 3 through 5 of the renal artery, meaning most nerves were located within 15 to 19 mm of the renal pelvis. This spatial distribution agrees with the finding of a previous study [23]. An advantage of MRgFUS over catheter-based renal denervation techniques is MRgFUS can be applied independent of vascular structure. In this study, the regions surrounding the renal pelvis were easily targeted. Although nearly the entire kidney was in the near field of the acoustic window, there was no damage detected in the kidney tissue at necropsy. In addition, the T1- and T2-maps measured no acute tissue changes in the kidney as a result of the renal denervation procedure.

While performing unilateral denervation allowed each animal to serve as its own control, the achievable reduction in NE ratio may be limited in this study compared to a bilateral denervation protocol. Bilateral denervation was not possible in this study because of the amount of gas present in the bowel and the prominent spinal transverse process, which limited the acoustic window accessible by the transducer to the right side of the animal. A different transducer design or other hardware modifications may have been able to access the left renal artery making bilateral denervation possible.

## Conclusions

This study demonstrates an increased efficacy effect in renal denervation using MRgFUS when higher in situ acoustic intensity levels are applied, as measured by a reduction in norepinephrine in groups treated at the higher acoustic intensity levels. The quantitative measurement of both T1- and T2-maps before and after the renal denervation treatment demonstrated that there was no acute injury in the kidneys, but edema was present in the muscle tissue in 63% of the animals (88% of the animals in which the T1 and T2 measurements were successfully acquired). These results indicate the need to incorporate patient-specific details in the treatment planning of MRgFUS renal denervation treatments to ensure both the safety and efficacy of the procedure.
